# Linkage Disequilibrium Decay and Past Population History in the Human Genome

**DOI:** 10.1371/journal.pone.0046603

**Published:** 2012-10-02

**Authors:** Leeyoung Park

**Affiliations:** Natural Science Research Institute, Yonsei University, Seoul, Korea; Aarhus University, Denmark

## Abstract

The fluctuation of population size has not been well studied in the previous studies of theoretical linkage disequilibrium (LD) expectation. In this study, an improved theoretical prediction of LD decay was derived to account for the effects of changes in effective population sizes. The equation was used to estimate effective population size (N_e_) assuming a constant N_e_ and LD at equilibrium, and these N_e_ estimates implied the past changes of N_e_ for a certain number of generations until equilibrium, which differed based on recombination rate. As the influence of recent population history on the N_e_ estimates is larger than old population history, recent changes in population size can be inferred more accurately than old changes. The theoretical predictions based on this improved expression showed accurate agreement with the simulated values. When applied to human genome data, the detailed recent history of human populations was obtained. The inferred past population history of each population showed good correspondence with historical studies. Specifically, four populations (three African ancestries and one Mexican ancestry) showed population growth that was significantly less than that of other populations, and two populations originated from China showed prominent exponential growth. During the examination of overall LD decay in the human genome, a selection pressure on chromosome 14, the gephyrin gene, was observed in all populations.

## Introduction

Linkage disequilibrium (LD) is an important parameter in population genetics. The LD in the human genome has been used to determine the association between variants and traits [1], and efforts to understand selection pressures have been based largely on the LD status of populations [2]–[5], The theoretical basis of expectations for LD was established by the pioneering efforts of theoretical geneticists [6]–[14]. Theoretical studies of LD, which deals with two variables, were more difficult than studying mutations, which dealt with one variable. There are several types of LD, but the most widely used is the squared correlation coefficient, r^2^. Initial effort on the expectation of LD was based on the diffusion approximations, which indicated that at equilibrium, the ratio of expected values in r^2^, σ_d_^2^, would reach approximately 1/(4Nc) when Nc was larger than 1, where N is the effective population size and c is the recombination rate [7]. Continued efforts enhanced the accuracy of the expectation of r^2^ based on the ratio of expected values or related improvements [10]–[12], [15]–[18].

Instead of using the ratio of expected values, a recurrence relation was derived for the direct expectation of LD based on a conditional probability of identity by descent at the second locus given identity by descent at the first locus. This approach resulted in the formula 1/(4Nc+1) for small values of c [16], [19], [20]. This formula was derived from the relation where the squared LD (r^2^) equaled the probability (Q) that two genes at a locus are identical by descent (IBD), given that two genes at a linked locus are IBD [19]. The recurrence relation is indicated below [20]. Including this one, all the previous methods assumed a constant effective population size. As indicated in a previous study [21], the population size of the previous and current generations influences the expected r^2^ value. Therefore, potential effects of the population size of the previous and current generation should be properly incorporated into a recurrence formula.
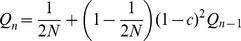



Recent advances in genomic technology have enabled the genome-wide observation of LD [22]–[24]. It has been suggested that past population history can be inferred from linkage disequilibrium [25]. Previous studies that estimated N_e_ based on LD identified recent human population growth [26]–[28]. However, in the method in which the estimation of N_e_ is based on chromosome segment homozygosity, the assumption of linear N_e_ with time did not work well with exponential growth or complicated population size changes [27]. In the estimation of N_e_ based on LD, the method was only applicable to small recombination rates due to approximation [26], [28]. The N_e_ estimates obtained in these studies did not represent the actual N_e_ changes at each generation, so it became necessary to simulate results regarding various situations that human populations might have experienced. In addition, the corrections made for sampling errors had significant bias, as noted in a previous study [29].

Despite its importance, there has been no study of the accurate theoretical expectation of squared LD with respect to the fluctuations in effective population sizes at each generation. In this study, a complete theoretical prediction of LD with fluctuating N_e_ was derived based on the recurrence formula from previous studies [19], [20]. This study investigated the expectation of LD decay involving changes of effective population size under various circumstances, and the results were applied to the human genome using HapMap phase III data to infer the past population history of each human population.

## Methods

### Theoretical expectation of LD decay in a finite population

If ideal Wright-Fisher population assumptions hold for a given population, variances due to genetic drift and recombination will be the main factors for the decay of linkage disequilibrium. As discussed in the previous study, the simplest random mating system; monoecious with selfing, was considered primarily [12], [21]. In this system, closed populations, discrete generations, and the absence of recurrent mutation were assumed. Let N_n_ be the population size at the n^th^ generation, and let N_n−1_ be the population size at the (n−1)^th^ generation. The population sampling procedure from one generation to the next can be described as follows: 1) sampling of 2N_n_ individuals from N_n−1_ with replacement; 2) generation of gametes from each individual among 2N_n_ individuals selected; and 3) random pairing of the gametes that were generated. The probability of each gamete differs depending on the recombination rate.

Considering that the probability that genes at both loci are identical by descent equals r^2^ [19], the influence of effective population size for the previous and current generations was incorporated into the recurrence formula from previous studies [20]. There are two samplings in the population sampling procedure: 1) a sampling of an individual from 2N_n−1_ gametes and 2) a sampling of gametes from a gamete pool generated from 1). The sampling of an individual from N_n−1_ means a sample of two gametes from the available pool, generated at the previous generation. Here, as in the previous study [19], sampling with replacement was assumed. When both haplotypes of the selected individual are IBD with a probability of 1/(2N_n−1_), recombination at the current generation does not change the IBD status. Therefore, an additional departure of LD, c^2^/(2N_n−1_), should be added for the portion of recombination. All the generated gametes are IBD, and a further sampling procedure provides no influence on the IBD status. For the gamete portion with no recombination, the proportion of recombinants in the probability, 1/(2N_n−1_), is 2c/(2N_n−1_). Therefore, the rest, 1−2c/(2N_n−1_), should be applied to the portion without recombination. The random sampling from the generated gamete pool consisted of 2N_n_ gametes produced the formula described in the Introduction. At this time, there is no further recombination. This approach finally leads to Eq. 1, in which r^2^_n−1_ is the LD of the previous generation, and N_n−1_ and N_n_ represent the effective sizes of the previous and current generations, respectively. 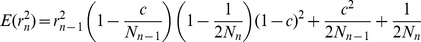
(1)


Eq. 1 was verified through simulations using various extreme values for N_n_, N_n−1_, r^2^_n−1_, and c (Table S1). The detailed simulation procedure is described in the following simulation section. As indicated in the previous study [19], the assumption for sampling with replacement involves a sample size that is not excessively small. For a similar reason, the equation could show expectations deviated from real data for the case of extreme allele frequencies with a small population size.
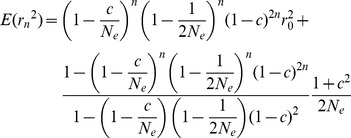
(2)


In Eq. 1, changes in the effective population size were reflected in the linkage disequilibrium. When N_e_ is constant, the general expression for n generations is Eq. 2. At equilibrium, r_n_^2^ becomes equal to the r_n−1_^2^, which can be expressed as (1+c^2^)/((1−(1−1/(2N_e_))(1−c/N_e_)(1−c)^2^)×2N_e_). This expression can be derived from Eq. 2, when the generation (n) approaches infinity. For a constant recombination rate, the expected linkage disequilibrium at equilibrium depended only on the effective population size. If the equilibrium is defined as occurring when the difference between r_n_^2^ and r_eq_^2^ became less than 1/(2N_e_), which is chosen because the r^2^ values at equilibrium are dependent on population size and the choice makes the equation simpler, the required n generation can be expressed as Eq. 3. As shown in Table 1, the time required to reach equilibrium differed depending on the recombination rate and the effective population size. As the effective population size became large and the recombination rate became small, more time was required to reach equilibrium. However, when the value of r^2^ at equilibrium is very high (N_e_ = 100 and c = 0.0001), the time required to reach equilibrium may be expected to decrease.

**Table 1:** The generation (age) until equilibrium (when the difference between generation becomes less than 1/(2N_e_)) depending on N_e_ and recombination rate (c)).

N_e_	100		500		1000		5000		10000	
c	age	E(r^2^)	age	E(r^2^)	age	E(r^2^)	age	E(r^2^)	age	E(r^2^)
0.5	4	0.00831	5	0.00167	11	0.00083	13	0.00017	14	0.00008
0.4	5	0.00902	7	0.00181	15	0.00091	18	0.00018	19	0.00009
0.3	7	0.01060	10	0.00213	21	0.00107	26	0.00021	28	0.00011
0.2	12	0.01427	15	0.00288	34	0.00144	41	0.00029	44	0.00014
0.1	24	0.02592	33	0.00529	72	0.00265	87	0.00053	94	0.00027
0.05	49	0.04892	67	0.01018	147	0.00511	179	0.00103	193	0.00051
0.01	201	0.20084	325	0.04785	717	0.02451	907	0.00500	979	0.00251
0.001	576	0.71440	2165	0.33345	4913	0.20008	8323	0.04764	9395	0.02440
0.0001	391	0.96154	4261	0.83334	10576	0.71430	44018	0.33334	64490	0.20001
From MAF*	269		1346		2692		13460		26920	

(*: mean allele age when minor allele frequency (MAF) is 0.4 [53]).



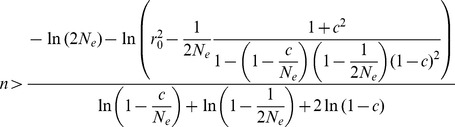
(3)


### Data

HapMap Phase III genotype data from the HapMap project, which included the original and expanded HapMap samples, were used for the estimations [22]–[24], [30]. The original HapMap samples were collected from four geographically diverse populations: Yoruba in Ibadan, Nigeria (YRI); Japanese in Tokyo, Japan (JPT); Han Chinese in Beijing, China (CHB); and CEPH (Utah, USA residents with ancestry from northern and western Europe, CEU). Additional samples were collected from seven populations: Maasai in Kinyawa, Kenya (MKK); Luhya in Webuye, Kenya (LWK); Chinese in metropolitan Denver, CO, USA (CHD); Gujarati Indians in Houston, TX, USA (GIH); Tuscans in Italy (TSI); African ancestry in the southwest USA (ASW); and Mexican ancestry in Los Angeles, CA, USA (MEX). The ASW, CEU, MEX, MKK, and YRI were family samples, and only the parents (indicated as ASWp, CEUp, MEXp, MKKp, and YRIp, respectively) were used in this data analysis. A total of 1198 samples were analyzed. For the accuracy of estimates, single nucleotide polymorphisms (SNPs) without missing data were used for analysis of the HapMap data.

### Simulation

Simulation was conducted with 1,000 pairs of diallelic loci for various population sizes based on the previous model. Starting from the complete LD (r^2^ = 1), only two initial haplotypes for each pair at the first generation were generated by a binomial draw based on allele frequencies of 0.5. The LD decay was based on a population sampling procedure similar to that used previously [21]. Let N_ep_ be the population size at the previous generation and N_ec_ be the size at the current generation. First, 2N_ec_ individuals were selected from N_ep_ individuals for mating. Second, one transmitting gamete was generated from each of the 2N_ec_ individuals. When the individual presented haplotypes such that its recombinants were distinguishable from the original haplotypes, whether the gamete would be a recombinant was determined by binomial draw based on the recombination rate. A gamete haplotype was then selected from two haplotypes, either recombinant or not. Finally, random pairings of the gametes generated N_ec_ individuals. This procedure was repeated for N generations. During simulations, frequent allele frequencies were maintained (≥0.1 and ≤0.9), by repeating population sampling with the frequencies below 0.1 or over 0.9.

When the allele frequency is extreme for a given population size, only several limited r^2^ values are available, which deviates from the expectation of r^2^. The maintenance of the allele frequencies within a certain range was arranged to avoid such a phenomenon. In reality, these simulation results did not show a distinguishable difference from simulation results with allele frequencies maintained between 0 and 1. All the simulations in this study were executed using the R statistical package with additional C++ coding of the core computation. The generation of every random number was based on the R statistical package.

**Figure 1:**
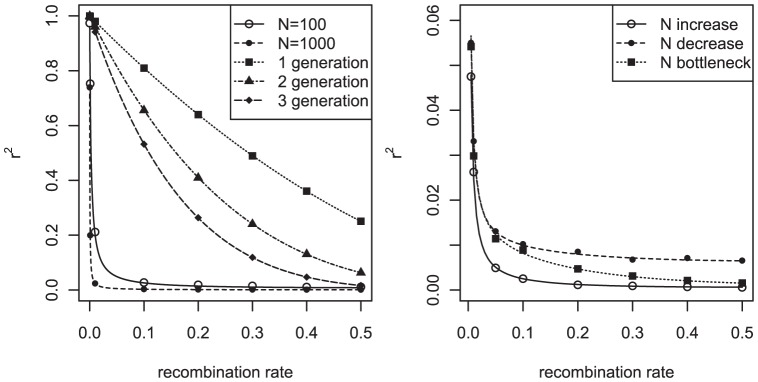
Comparisons between theoretical (line) and simulated (dot) r^2^. (A) N_e_ constant: for constant N_e_ = 100 and 1000, the r^2^ values at equilibrium decayed from the complete LD are plotted. The 1, 2, and 3 generations indicate r^2^ values with the decay generation(s) from the complete LD when N_e_ equaled 1000. (B) N_e_ changes: After reaching equilibrium with a constant N_e_ of 1000, three different circumstances of population size were applied for five generations, i.e., increments (1000,1100,1200,1300,1400), decrements (1000 ,500,300,200,100), and bottlenecks (1000,100,100,1000,1000).

The simulation results were exactly matched with the equations. As shown in Figure 1, when linkage disequilibrium decayed from the complete linkage disequilibrium value of 1, the simulation results (dots) corresponded well with the expected values (lines) derived from Eqs. 1 and 2. The generations at equilibrium were derived from Eq. 3, and the simulated values showed excellent correspondence with the theoretical equilibrium value, even reflecting the difference of 1/(2N_e_), as shown in Figure 1A. To examine LD as the population size changed, the three situations that were most likely, i.e., increment, decrement, and bottleneck of population size, were selected. As shown in Figure 1B, the results indicated excellent agreement between the theoretical and simulated values for all three situations.

### Sampling and errors

Sampling induces additional departures to the expected r^2^ value [12]. When the r^2^ value of the original population was zero and the haplotype frequencies were estimated using maximum likelihood methods, the sampling variance becomes 1/n_s_+1/(2N_e_), where n_s_ is the sample size and N_e_ is the original population size [10], [12], [21], [25]. When the expected linkage disequilibrium was not zero and the direct identification of haplotypes was possible, the amount of sampling variance due to r_o_^2^ was reduced by a factor of (1−r_o_^2^), where r_o_^2^ was the original LD before sampling. Eq. 1 simply yielded the expected r^2^ as (1−1/2n_s_)r^2^_0_+1/2n_s_, assuming no recombination. This result is consistent with previous studies [19], [20], where the probability that two genes at both loci are IBD for a selected individual in a sampled population provided the same r^2^ expectation. For a sampled population, the probability that both genes descend from the same gene is not dependent on the original population size but rather on the sampled population size.

This expectation is applicable when the direct identification of haplotypes is available. The maximum likelihood estimation of haplotype frequencies induces additional departures similar to the case of linkage equilibrium [10], [12], [21], [25]. The sampling variances from the likelihood estimation of haplotype frequencies showed almost the same trends that r_o_^2^ reduced by a factor of (1−r_o_^2^), as the variances obtained from the direct identification of haplotypes, with small mean squared errors based on simulations (Table S2). Increasing the sample size reduced the mean squared error. Therefore, the expectation of linkage disequilibrium due to sampling can be expressed approximately as Eq. 4. Here, r_s_^2^ indicates the linkage disequilibrium of the sampled population. When linkage disequilibrium was not zero and haplotype frequencies were estimated using maximum likelihood methods, an accurate equation was difficult to obtain [10]. Since Eq. 4 fit relatively well, the current study utilized Eq. 4 to obtain r^2^ values for the original populations.
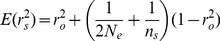
(4)


For simulations, the sampling of n_s_ individuals among N_ec_ individuals with replacement was conducted after the population sampling procedure described above. The allele frequencies of the original population were 0.5, and the r^2^_0_ values were based on fixed haplotype frequencies. The haplotype frequencies for two loci were estimated from the sampled genotypes based on the expectation maximization (EM) algorithm. To examine the difference between the original r^2^ and the sampled r^2^, the mean squared errors were obtained from simulations for a population size of 1,000 and various sample sizes (Table S2).

### LD and N_e_ estimation

The LD was measured with distances in each chromosome for each population in the HapMap data, and only autosomes were examined. In samples with an insufficient sample size, SNPs with small minor allele frequencies resulted in r^2^ values deviated from expectations, due to limited values in the frequencies of haplotypes that consist of minor alleles. To avoid extreme values that impede the correct expectation of r^2^ at equilibrium, SNPs with minor allele frequencies (MAF) higher than 0.4 were used for estimating r^2^. In addition, the LD between aged SNPs at equilibrium should be used for N_e_ estimation. SNPs with high minor allele frequencies would be a reasonable choice for aged SNPs. The haplotype frequencies were derived using the EM algorithm.

In each chromosome, the distances between variants were separated as a unit of 10,000, and the mean of r^2^ estimates in each unit were used to examine LD decay depending on recombination rates in the human genome. Recombination rates varied depending on genomic region [31], but it was clear that there was an apparent correlation between distance and recombination rate. In addition, the population differences observed in a recent study, in which fine-scale recombination rates were estimated based on the Icelandic genealogy database [32]. Therefore, the recombination rates were derived directly from the distances between variants. The Haldane map was used to convert distances to recombination rates, and 1 Mb was considered to represent approximately 130 cM [33].

Assuming that the effective population size has been constant, N_e_ estimates with respect to recombination rates were obtained, which enabled inferences regarding past changes of effective population size. For the calculation, the average r^2^ of all chromosomes except chromosome 14 was used because selection pressures were observed in a large region of chromosome 14 for all populations (see Results section). Since the expression of the sampling variance involved the current effective population size, the current effective population size should be estimated first, based on deviations from the Hardy-Weinberg equilibrium [21]. Relying on Eq. 4, the r^2^ of the original population (E(r^2^) in Eq. 5) was obtained and used to find N_e_ from Eq. 5 by solving a cubic equation derived from Eq. 1, assuming constant N_e_ except for the current generation. Thus, the r^2^ at the previous generation became (1+c^2^)/((1−(1−1/(2N_e_))(1−c/N_e_)(1−c)^2^)×2N_e_). In Eq. 5, N_e_ is the constant effective population size, N_c_ is the current effective population size, and c is the recombination rate. For theoretical predictions for various situations, the same method was applied using the known N_c_.
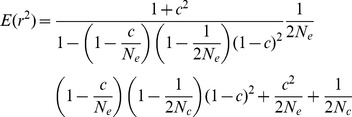
(5)


### Descriptions of various population histories for theoretical predictions

To infer past population history from patterns of LD according to recombination rates, three basic changes in population size, increment, decrement, and bottleneck were examined, and simulations were conducted based on possible human population histories. For the human population histories, the overall world population history was first examined and then three important events, increments, decrements and bottlenecks, were further investigated. Because the N_e_ estimates reflect the recent population history much more than the old population history, the recent population history was examined in priority.

#### Three basic changes in population size

**Figure 2:**
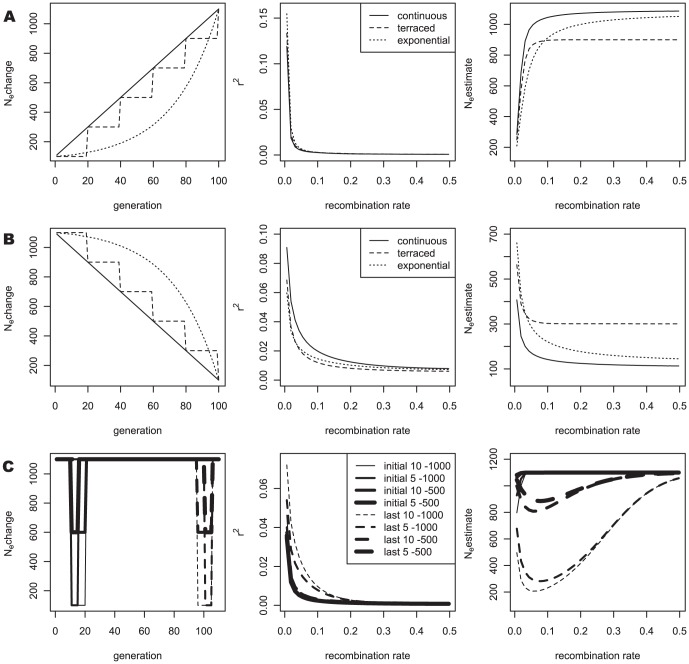
Three basic changes in population size and the differences in r^2^ and N_e_ estimates. (A) Continuous, terraced, and exponential increment; (B) Continuous, terraced, and exponential decrement; (C) Variable bottlenecks based on time, duration, and amount of reduction.

Because the ancient population histories were not very influential in the N_e_ estimates for recombination rates (higher than 0.0065), the most recent 100 generations became the focus. For both increment and decrement, three different situations, continuous, terraced, and exponential, were studied (Figure 2). The population size changed from 100 to 1100 or vice versa, and the changes for 100 generations were started when the r^2^ of the population reached equilibrium for a given initial population size (from Eq. 3). The continuous increment or decrement involved 10 increments or decrements per generation for 100 generations. In the terraced increment, size changes occurred five times for 100 generations, with a rate of 200 per generation. For the exponential changes, the population size increased or decreased exponentially with a base of 1.0366 for 100 generations (N_n_ = N_n−1_+1.0366^n^; n: 1∼100; the exponential part was rounded). The bottleneck was studied for two different terms of time, duration, and amount of reduction. Therefore, eight different bottlenecks were examined with a population size of 1100: 1) bottleneck for 1∼10 generations with a population size of 100; 2) for 1∼5 generations with a size of 100; 3) for 1∼10 generations with a size of 600; 4) for 1∼5 generations with a size of 600; 5) for 86∼95 generations with a size of 100; 6) for 91∼95 generations with a size of 100; 7) for 86∼95 generations with a size of 600; and 8) for 91∼95 generations with a size of 600.

#### Real world population history

The global population change from BC 10,000 to AD 2000 [34] was used. Population sizes for the years from 1970 to 2000 were estimates [34]; these estimates fit well with actual population changes and were used in the current study. The average length of human life was less than 36 years before the 18^th^ century but has increased in recent years [35]. Two thousand years ago in Rome, life expectancy was only 22 years. Therefore, differential generation times were applied. Specifically, until AD 500, the generation time was assumed to be 20 years; from AD 500–1400, 22 years; from AD 1400–1900, 25 years; and from AD 1900–2000, 30 years, thus making the total generations for the period from BC 10,000 to AD 2000 result in a sum of up to 4,989 generations (Figure S1). For N_e_, the million unit was omitted so that 200 million was considered as 200 for effective population size.

#### Exponential growth of effective population size

**Figure 3:**
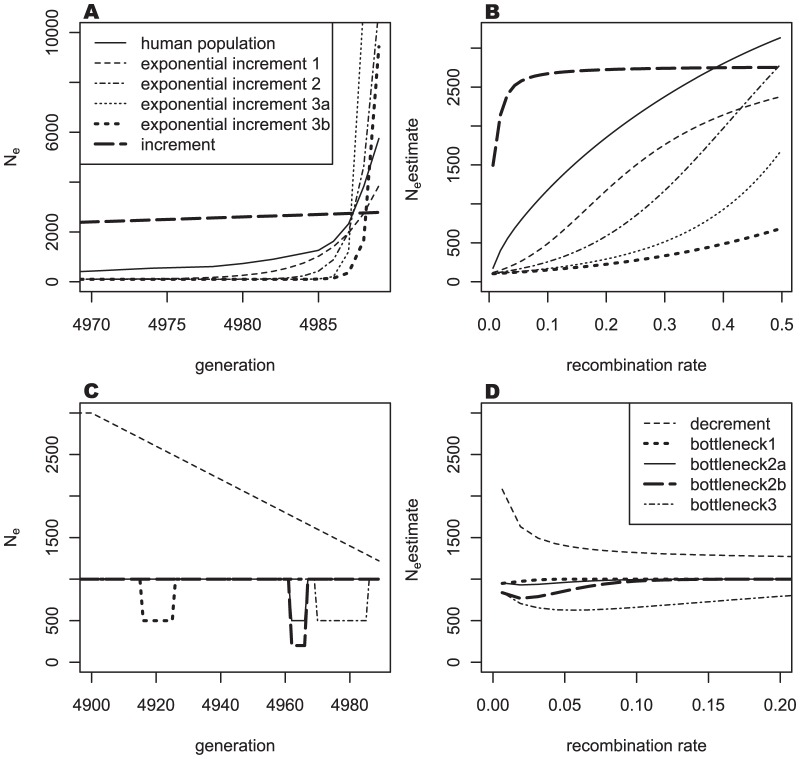
Past population changes and N_e_ estimates for various situations. (A) Various increments of population size; (B) N_e_ estimates based on (A); (C) A decrement and various bottlenecks in population size; (D) N_e_ estimates based on (C).

To more closely examine the impact of the recent exponential growth in human population on the estimate of effective population size, three different exponential growths in the most recent generations were modeled. For the first, a constant population size of 100 was sustained for 4969 generations; the population was then exponentially increased with a base of 1.42 for 20 generations, giving a final population size of 3851. The second model featured a constant population size of 100 for 4979 generations; the size of the population was then increased exponentially with a base of 2.4 for 10 generations, giving a final population size of 10,968. In the third model, two different increasing situations were applied. In the first situation, a constant population size of 100 was applied for 4984 generations; the size was then exponentially increased for five generations with a base of 10, producing a final population size of 111,210 (exponential increment 3a in Figure 3). In the second situation, the population size was exponentially increased for the last five generations with a base of 6, yielding a final population size of 9430 (exponential increment 3b in Figure 3).

#### Continuous increment and decrement of effective population size

A continuous increment for the last 89 generations was modeled after a constant population size of 1000. The increment size was 20 per generation. For the model of decreasing effective population size, a constant population size of 3000 was maintained for 4900 generations, and the decrement was continued for 89 generations. The amount of decrement was constant at 20 per generation.

#### Bottleneck of effective population size

In real human population history, several bottlenecks have occurred. To examine the impact of bottlenecks on the estimates obtained in this work, bottlenecks in three different periods were modeled. One period included generations 4916 to 4925 starting at approximately AD 500 (Bottleneck 1 in Figure 3). Another bottleneck period included generations 4962 to 4966 in the 13^th^ century [36], when the black death raged (Bottleneck 2a in Figure 3). Since more recent bottlenecks would have a greater impact on the N_e_ estimates, the bottleneck for five recent generations (4970∼4985) was modeled (Bottleneck 3 in Figure 3). In the model, the original population size was 1000, and a 50% reduction was modeled for these bottlenecks. To examine the effects of a greater reduction in population, the second bottleneck was repeated with an 80% reduction (Bottleneck 2b in Figure 3).

## Results

### LD expectation depending on recombination rates and N_e_ changes

The past N_e_ change can be inferred from the changes in N_e_ estimates according to recombination rates. Table 1 shows the time to equilibrium when constant population size was assumed. Smaller effective population size leads to faster equilibrium. More importantly, as the recombination rate became smaller, the LD between polymorphisms decayed more slowly so that the r^2^ value represented the changes of effective population sizes for a longer period of time (Table 1). Assuming constant population size, the estimates of N_e_ reflected past changes of effective population sizes, among which the more recent changes were more highly reflected. Depending on recombination rate, each N_e_ estimate represented N_e_ change in a different period of time. The N_e_ estimates derived from smaller recombination rates represented longer past changes of effective population size. Therefore, approximate inferences regarding past N_e_ changes were acquired from the estimates depending on the recombination rates.

Figure 2 shows the differences in r^2^ values and N_e_ estimates depending on the recombination rates for the various changes in population sizes. The N_e_ estimates show clear differences for population size changes, even when the r^2^ values show no big differences. Continuous increments in population size gave almost constant N_e_ estimates after steep increments at very small recombination rates. The terraced increment showed smaller N_e_ estimates at large recombination rates than did the continuous increment. Compared to the continuous increment, the exponential increment showed smoother N_e_ increments, as the recombination rate increased. The same trend was shown for the decrement. Similar to the continuous increment in population size, a continuous decrement in population size gave almost constant N_e_ estimates after steep decrements at very small recombination rates. The bottlenecks for the 1∼10 generations were not effective for the N_e_ estimates when the recombination rates were greater than 0.1, but the recent bottlenecks for the 86∼95 generations showed large differences at higher recombination rates. This result indicates that the earlier bottleneck might not be detectable in these N_e_ estimates and thus we need a detailed examination of N_e_ estimates in extremely small recombination rates. The size of the change in the bottleneck influenced the amount of reduction in N_e_ estimates, depending on the recombination rate.

Figure 3 shows N_e_ estimates for various situations according to human population history. Since the main interest in the current study was the human genome, N_e_ changes relevant to the history of the human population were examined intensively. Figures 3A and 3B show several exponential increments of population size, including a circumstance based on the real-world human population history [34]. Changes in actual human population size showed a steep increment in N_e_ estimates depending on recombination rates. As observed in Figure 2, there is a clear exponential increment during recent generations. To explain the N_e_ estimates based on the HapMap data, three exponential increments that differed in the period of exponential increment, i.e. the last 20, 10, and 5 generations, were examined. As the start point of an exponential increment approached the current generation, the increment in the N_e_ estimate became steeper at small recombination rates. The magnitude of bases influenced the shape at large recombination rates. As the recombination rate increased, the exponential increment 3a with an exponential base of 10 showed a steeper increment than the exponential increment 3b with an exponential base of 6.

Figures 3C and 3D show that the decrement of N_e_ decreased the N_e_ estimates as the recombination rate increased and that bottlenecks caused reductions in the N_e_ estimates at certain recombination rates. The N_e_ estimates could be changed depending on the time and amount of the decrement. The impacts of a bottleneck differed depending on the time, duration, and amount of reduction from the original population, as in Figure 2. The time at which the bottleneck began affected the reduction of the N_e_ estimates at certain recombination rates, and as the duration of the bottleneck increased, its impact on the reduction of the N_e_ estimate lasted longer. The amount of reduction from the original population directly determined the extent of reduction of the N_e_ estimates.

### LD decay and past population history in the human genome

**Figure 4:**
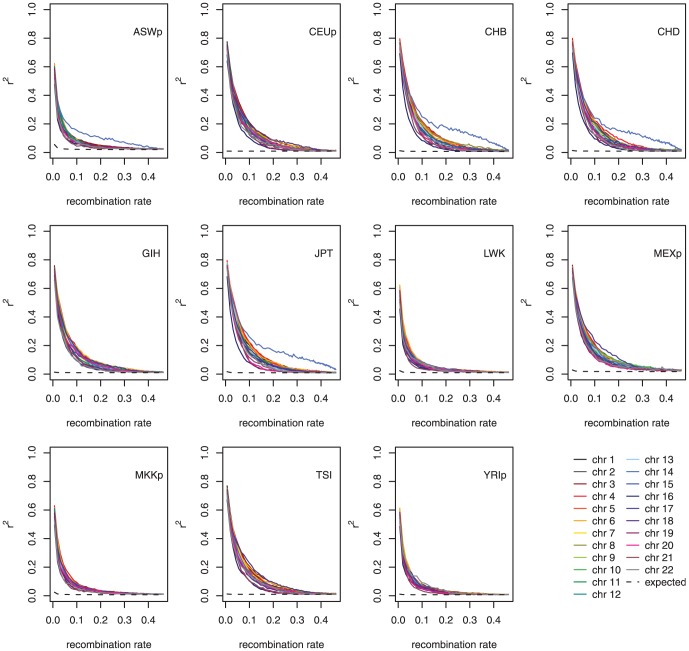
LD decay of the human genome depending on recombination rates. (Dashed lines represent the expected r^2^ at equilibrium assuming constant N_e_ with the N_e_ estimate of the current generation).

The theoretical expectation derived in the current study was applied to the LD in the human genome using HapMap data. Figure 4 indicates the LD decay in the human genome based on recombination rates, which can be obtained by converting distances. Each chromosome is indicated by a different color; most of the chromosomes showed similar LD decay overall. The dashed line in Figure 4 indicates the expected r^2^ at equilibrium when the effective population size is constant and the same as the estimate of current effective population size. It is clear from the figure that all the populations had larger current than past effective population sizes.

Four populations, ASWp, CHB, CHD, and JPT, showed increases in the r^2^ estimates in chromosome 14. When the positions of variants with high r^2^ values were examined in the four populations, all the variants in the regions from approximately 65,700 kb to 66,800 kb had high LDs between them, creating a large linkage disequilibrium block (Figure S2). Using variants with all frequencies, the same regions in all the other populations showed a similar LD block. Since the current study only used variants with a minor allele frequency (MAF) higher than 0.4, variants with all frequencies were examined within the specified region. By examining the frequency spectra of the region from 65,500 kb to 67,500 kb, extraordinary spectra were found for all populations; in these spectra the variants with specific frequencies occurred much more often than the variants with other frequencies (Figure S3).

The foregoing phenomenon was expected when selective sweeps occurred in a specific genomic region [2], [5]. This region had not been previously identified as a major region of selective pressure. This specific LD block region in chromosome 14 harbors the complete gephyrin gene (*GPHN*) (Figure S2). This gene encodes a multifunctional protein that catalyzed the molybdenum cofactor biosynthesis in the liver, kidney, and other non-neuronal organs and that plays a role in the postsynaptic targeting and clustering of glycine and GABA_A_ receptors at inhibitory synapses [37]. The gephyrin gene had a complex intron-exon structure and multiple splice sites. Isoforms of the protein are expressed in a tissue-specific manner and with apparent species-specific differences [37]. The center of the LD block was located within intron 2 of this gene.

**Figure 5:**
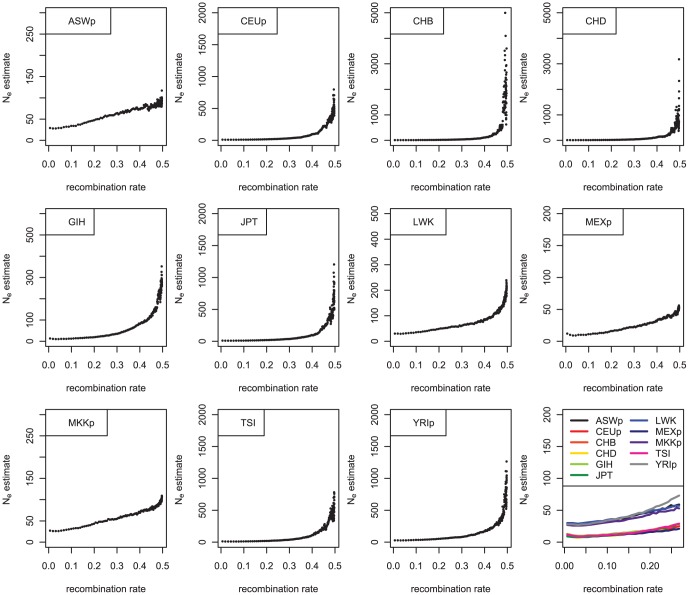
N_e_ estimates of human population samples depending on recombination rates.

As shown in Table 1, the time required to reach equilibrium differed depending on recombination rate and effective population size. Over time, the population sizes had been changed, and the LD, which depends on recombination rate, can indicate past changes in effective population size. The effective population size was estimated depending on the recombination rates. In this estimate of N_e_, the current effective population size was not involved. Figure 5 shows that all the populations experienced recent exponential increases in population size, confirming the previous results [27]. From the results shown in Figures 2 and 3, it can be concluded that this exponential increment occurred specifically in most recent generations, probably less than five generations before the current generation; this finding is reasonably acceptable considering the recent explosive growth in the human population.

Two distinctive patterns are illustrated in Figure 5. One is the occurrence of an extreme exponential increment in several very recent generations, shown in CEUp, CHB, CHD, GIH, JPT, TSI, and YRIp; the other is a less extreme exponential increment in the effective population size, shown in ASWp, LWK, MEXp, and MKKp. The former populations showed exponential increments with extremely large magnitudes, whereas the latter populations displayed increments of much smaller magnitudes. The major differences between these two groups result from a very recent, more explosive population growth in one group. When the N_e_ estimates for small recombination rates (less than 0.3) were examined, all four populations of African descent, ASWp, LWK, MKKp, and YRIp showed slightly larger N_e_ estimates than those of other populations, suggesting that long ago their population sizes were larger than those of other populations (see the last part of Figure 5 and Figure S4.) This result supports the occurrence of an out-of-Africa expansion.

It was noteworthy that of the populations with milder past growth, one was of African descent, two were African populations, and one was of Mexican ancestry. Among the populations with African ancestry, only YRIp showed a pattern that was similar to other populations. It is clear that suppressive factors impeded population growth in these four populations in comparison to others. As shown in a previous study [21], the current results also indicate that the recent immigrant populations show more similar trends in their countries of origin than in the countries to which they immigrated (CHD, GIH, and MEXp). These results are not surprising because these samples were collected from individuals who identified themselves as having at least three out of four grandparents belonging to their original population. As illustrated in the panel of Figure 5, bottlenecks were observed in many populations, including CEUp and TSI at the approximate time when the black death occurred [36]. However, the effects of these bottlenecks were relatively minor compared to the exponential growth.

### Past population history derived from the genome and historical studies

The N_e_ estimates obtained using HapMap data effectively represent the recent extreme exponential growth in the human population. These results verify previous observations of human population growth [26], [27] and provide further detailed information regarding the individual population histories. Four populations, i.e., ASWp, LWK, MEXp, and MKKp, showed mild exponential growth in effective population size, and CHB and CHD showed prominent exponential growth. Among the four population samples of African descents, YRIp was the only population, showing population growth similar to most other populations. To verify these results, historical reports were examined in this section.

Historically, the slave trade and colonial invasion impeded population growth among Africans in recent centuries [38]. In this region, independence from colonial domination began after World War II ended in 1945 [39]. Both LWK and MKKp are Kenyan and thus might be expected to be relatively less affected by the initial slave trade due to regional effects; however historians have suspected that there was at least a slight population reduction in East Africa during the colonial domination period [38]. The current results partially support this idea and suggest that East Africa did not experience such recent explosive population growth that occurred on most other continents.

Ibadan is located on the West African coast near the Bight of Benin, a central historical port for the slave trade. However, YRIp showed population growth more similar to that of CEUp and TSI than to that of LWK and MKKp. In the early 18^th^ century, Oyo, the Yoruba kingdom located near Ibadan, was an important supplier of slaves, who were mostly acquired in the conquest of surrounding countries [38], [40]. This expanded the money supply and brought trading activities into the region [38], [40], which might have augmented the population growth, making it similar to the growth on other continents.

During the colonial domination period in Africa, most women were married as soon as they reached child-bearing age, and polygamy was relatively common until recently [38]. It is also likely that the average lifetime in Africa during this period was shorter than on other continents. It therefore follows that the time per generation was shorter in Africa than on other continents. On most continents, if a steep population increase occurred during the last 100 years, this increment occurred over three to four generations. However, for Africans, the increment might have occurred over four to five generations, and this should be considered in the interpretations of relevant samples. The relatively early starting points of the exponential growth in four populations of African descent in the last panel of Figure 5 might be explained by this difference.

The ASWp consists of people of African descent who, for the most part, were moved involuntarily to the U.S. due to the slave trade [41]. During their transportation to America, only half the enslaved population survived and remained available for work [38]. Although slavery was abolished in the U.S. in 1865, apparent racial discrimination persisted until 1964 [41], which could have been an influence on the relatively impeded population growth of this population. People in the MEXp sample had Mexican ancestry. Mexicans suffered a recent bottleneck after the colonial invasion, and the population was not restored to half its size at the time of the voyage of Columbus until the late 18^th^ century [42]. Therefore, the relatively small increments observed in both ASWp and MEXp in Figure 5 would be reasonable, but strong bottleneck effects were not observed in these populations compared to other populations.

Polygamy reduces the effective population size [43]; this may partly explain the results with LWK and MKKp, in which the culture persisted up to date. In addition, there might be an unknown effect in admixed populations such as ASWp and MEXp [44]. However, it is obvious that the results obtained with these four population samples indicate that their population growth rate in recent generations has been much smaller than that of other populations, and these results correspond with historical studies.

CHB and CHD were prominent among samples with extreme exponential growth. China has experienced almost continuous population growth from 1400 to the present, except for the period from 1683 to 1700, during which a population decrement occurred [45]. China is currently the world's most populous country. Thus, extreme exponential growth could be expected in the population samples originally from China. Colonial invasion of China began in the late 19^th^ century and lasted for a very short period. In India, colonial invasion began in the late 18^th^ century; however the colonization of India was much more cautious and very different from that of Africa and America [46]. The length of the colonial period in India was approximately 100 years longer than in China; this might explain why the population growth of GIH was not as steep as that of CHB and CHD, even though India is the second most populous country in the world.

## Discussion

The current study provides an actual expression of the expectation of LD decay depending on recombination rate and changes in effective population size. The theoretical expectation derived in this study showed an accurate agreement with simulation results. In addition, this study provides an advanced solution for sampling variances in LD estimations. Using the expression, the past population history can be derived from LD data. Naturally, the recent population history has a greater influence on the N_e_ estimates than does the old population history. Applications to the human genome showed good agreement with the changes in human population that have occurred in relatively recent generations. By enhancing the accuracy of r^2^ expectation, the current study appears to be useful for studying the patterns of linkage disequilibrium in relation to changes of effective population size, providing a better understanding of past population histories.

However, it should also be noted that the current method provided only an approximate picture what type of population changes the sampled population had experienced, especially with respect to recent generations. For better inferences, a model fitting based on a likelihood could be helpful. Because smaller recombination rates need more time to reach equilibrium, the mean linkage disequilibrium of small recombination rate would involve a wider range of ancient population changes. If the allele ages could be estimated accurately and the inaccuracies due to extreme frequencies and selection pressures were appropriately adjusted, a serial estimation of the effective population size from current to ancient generations might be possible. In addition, the method is based on the assumption of a closed population, which is not true in the real world. Migrations should also be considered for the application to real populations. In that case, a more accurate picture of past population histories could be presented.

The selection pressure on chromosome 14 found in this study was unexpected. Although the LD block was larger than 1 Mb, previous studies that attempted to identify regions particularly affected by selection pressures in the human genome did not detect this region in a selective sweep using the HapMap data [24], [30], [47], [48]. Earlier studies did not list the region because it did not seem significant enough in their result tables representing extensive data analyses. One study, which used HapMap data, listed this region as under strong selection pressure in supplementary information that provided a summary of the strongest regions of selection in Europeans [49]. Another analysis, which used the Perlegen data, did not identify *GPHN* as a gene under strong selection pressure, but supplementary information indicated that this gene region fell within the significant selective sweep with a p-value of ∼0.0006 in the Chinese sample [4], [50]. In European-American and African-American samples, the p-values of ∼0.10 and ∼0.83, respectively, associated with this region were not significant [50]. A study aimed at identifying positively selected genes by comparing human and chimpanzee genomes showed no evidence for selection pressure on *GPHN* [51], presumably indicating that the selection pressure on this gene might have appeared relatively recently. Inconsistent results between these methods for finding selection pressures were well indicated in a previous review [52]. Further studies are necessary to elucidate the understanding of the selection pressures in this region and to assess the population differences in more detail.

In the current study, the theoretical expectation for change in effective population size was exactly matched with the simulation results. In the sampling expectation derived from Eq. 4 in the Methods section, the mean squared errors of the expectation after sampling were quite small. However, detailed simulation studies indicated that there was a more complicated relationship between the population LD, r_o_^2^, and the sampling variances than suggested by Eq. 4. When direct acquisition of haplotype frequencies without likelihood estimation was tested, the sampling estimates provided the exact relationship between r_o_^2^ and the variances. Therefore, either the development of an accurate expectation for the sampled genotype data or the direct identification of haplotypes is necessary for solving the problem of residual sampling variances. Since the errors were relatively minor and influenced the data equally, the results of the current estimation might not differ greatly.

Even considering this slight inaccuracy in sampling expectations, the current study provides a much more accurate expectation of LD for sampling than previous studies [26]–[28]. Previously, the reduction of sampling variances was not counted when the original LD was not zero, which may have serious bias into the results. The uncorrected sampling bias inflated N_e_ estimates at small recombination rates, especially 10,000∼20,000 years ago in their timeframe. The large N_e_ (up to several thousand individuals) at small recombination rates might result from this bias. In the present study, the bias was largely corrected, and very small N_e_ values (less than 50) was observed for very small recombination rates. The current results are more reasonable because in the past most people lived in very small communities and available mating was quite limited due to limited transportation systems.

Previously [26]–[28], N_e_ estimates were considered as the N_e_ at 1/(2c) generation ago. However, as shown in Table 1, the estimates merely represented the past N_e_ changes over time until equilibrium. Neither was the value for the generations until equilibrium equivalent to 1/(2c) nor did the estimates indicate the N_e_ at a certain number of generations in the past. Therefore, this approximate timeframe, 1/(2c), seems not appropriate, and simulations or other methods should be accompanied to infer the past population history properly. In addition, the previous studies were based on approximations that are applicable only to small recombination rates, suggesting that their observations on recent population increments might be biased as well. On the contrary, the current method is accurate for any recombination rate, when the population size is not too small and the frequencies are not extreme.

In this study, variants with MAF higher than 0.4 were used, and most of the variants were of sufficient age to have reached equilibrium, as shown in Table 1. However, for N_e_ estimates at very small recombination rates, the allele ages might not be sufficient. The smallest recombination rate used in this study was 0.0065, and most of the recombination rates used in this study appear to be safely within the range to result in equilibrium for variants with MAF greater than 0.4. The LD expectation in this study assumed decay from the complete LD value of one between the frequent variants. In most cases, the variants started from single mutation events, and the starting LD (r^2^) was not one unless they occurred on the same haplotype at the same time. Nevertheless, most aged variants at equilibrium would still provide valid estimates reflecting the N_e_ changes for different lengths of generations depending on the recombination rates.

In summary, the current study provides the first actual LD expectation regarding changes of effective population sizes. This advanced LD expression enables a more accurate inference of past N_e_ changes. In addition, this study provides a more accurate method of correcting sample size effect when LD is not zero. The application of the method to human genome data gave good agreement with the actual population changes documented in historical studies. This method provides a simple and accurate picture of the population changes the sampled populations had experienced, which is applicable to other populations of interest. The results of this study confirmed that LD in the genome would be a useful source for obtaining a detailed population history.

## Supporting Information

Figure S1 **The reconstituted population history based on generation time.** (A) from BC 10,000 to AD 2000; (B) an enlargement of (A) from AD 0 to AD 2000.
(PDF)

Figure S2 **The LD plot for the region from 65,500 kb to 67,500 kb in chromosome 14 using CHB data (data with missing genotype less than 1% and no monomorphic site).**
(PDF)

Figure S3 **The histogram of minor allele frequency for the region from 65,500 kb to 67,500 kb in chromosome 14.**
(PDF)

Figure S4 **The enlargement of the last figure of** **Figure 4** **; N_e_ estimates of human population samples depending on recombination rates up to 0.3.**
(PDF)

Table S1 Verification of Eq. 1 by simulations using extreme values.
(XLSX)

Table S2 Mean squared errors of sampling from fixed r^2^ for various sampling sizes with 10,000 SNP pairs and a population size of 1000 (r^2^_o_: the original r^2^ of a population; ss: sample size).
(DOC)
