# Correction: Linkage Disequilibrium Decay and Past Population History in the Human Genome

**DOI:** 10.1371/annotation/2dffa2d3-6cc9-4393-8b54-70e0888733f2

**Published:** 2012-11-08

**Authors:** Leeyoung Park

There were errors in the Funding section. The correct funding information is as follows: This work was supported by the Korea Research Foundation Grant funded by the Korean Government (MOEHRD) (KRF-2007-532-C00017) and by the National Research Foundation of Korea Grant funded by the Korean Government (NRF-2009-353-C00061). Supercomputing resources were supported by the Korea Institute of Science and Technology Information (KISTI) (KSC-2010-S00-0016 & PLSI). The funders had no role in study design, data collection and analysis, decision to publish, or preparation of the manuscript. 

