# Supplementary figures and images for: Linkage Disequilibrium Decay and Past Population History in the Human Genome
Source: PLoS One. 2012 Oct 2;7(10):e46603. doi: 10.1371/journal.pone.0046603 (PMC3462787; doi:10.1371/journal.pone.0046603)

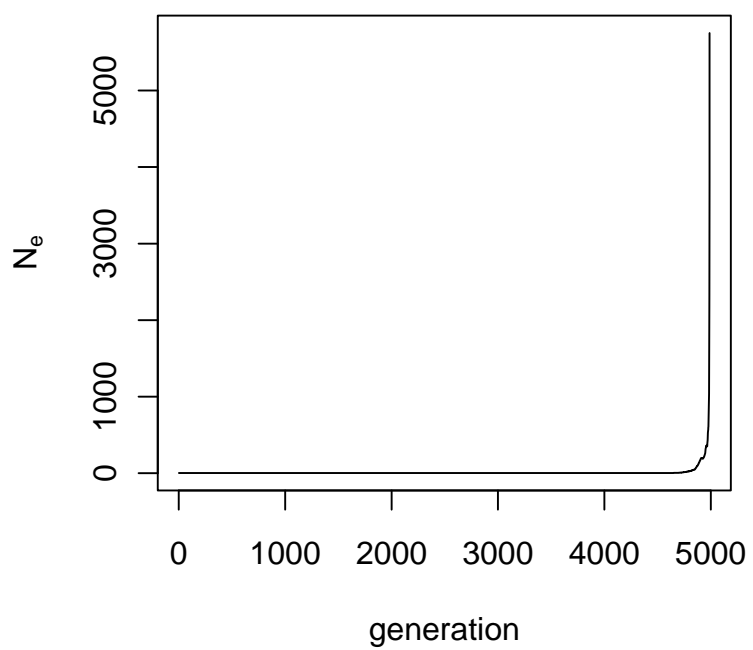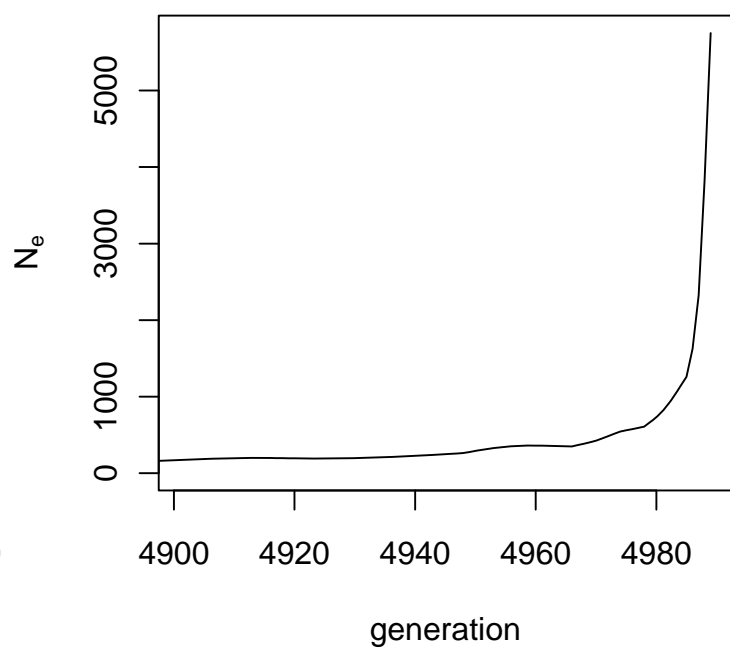

Supplement: Figure S1 — The reconstituted population history based on generation time. (A) from BC 10,000 to AD 2000; (B) an enlargement of (A) from AD 0 to AD 2000. (PDF) [file pone.0046603.s001.pdf]

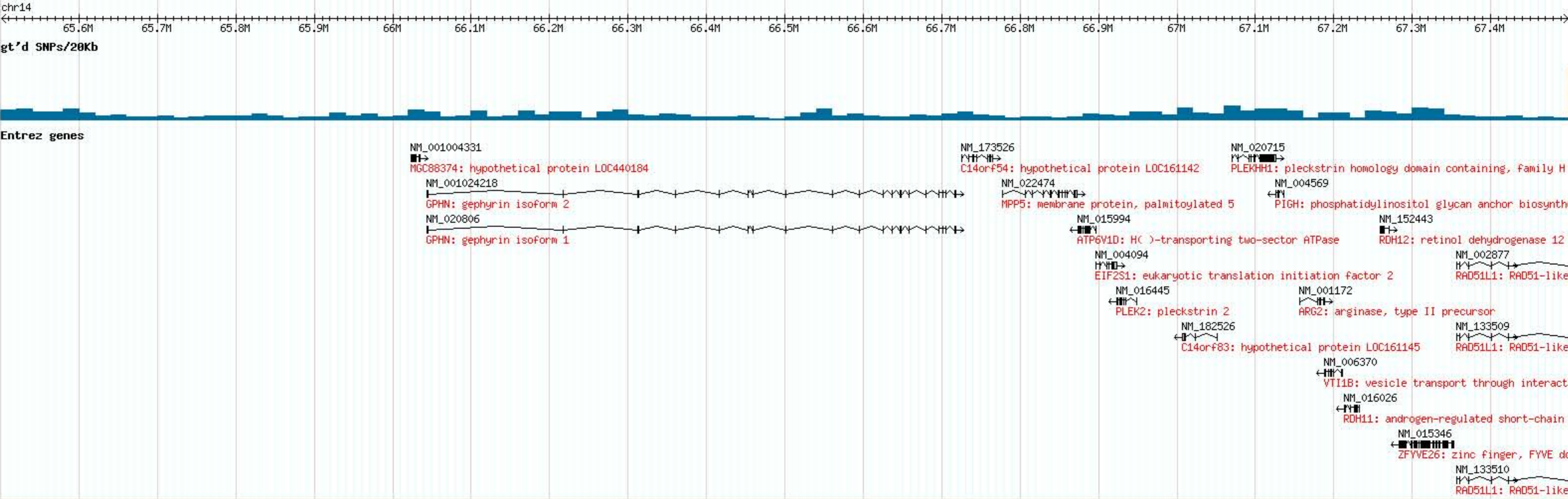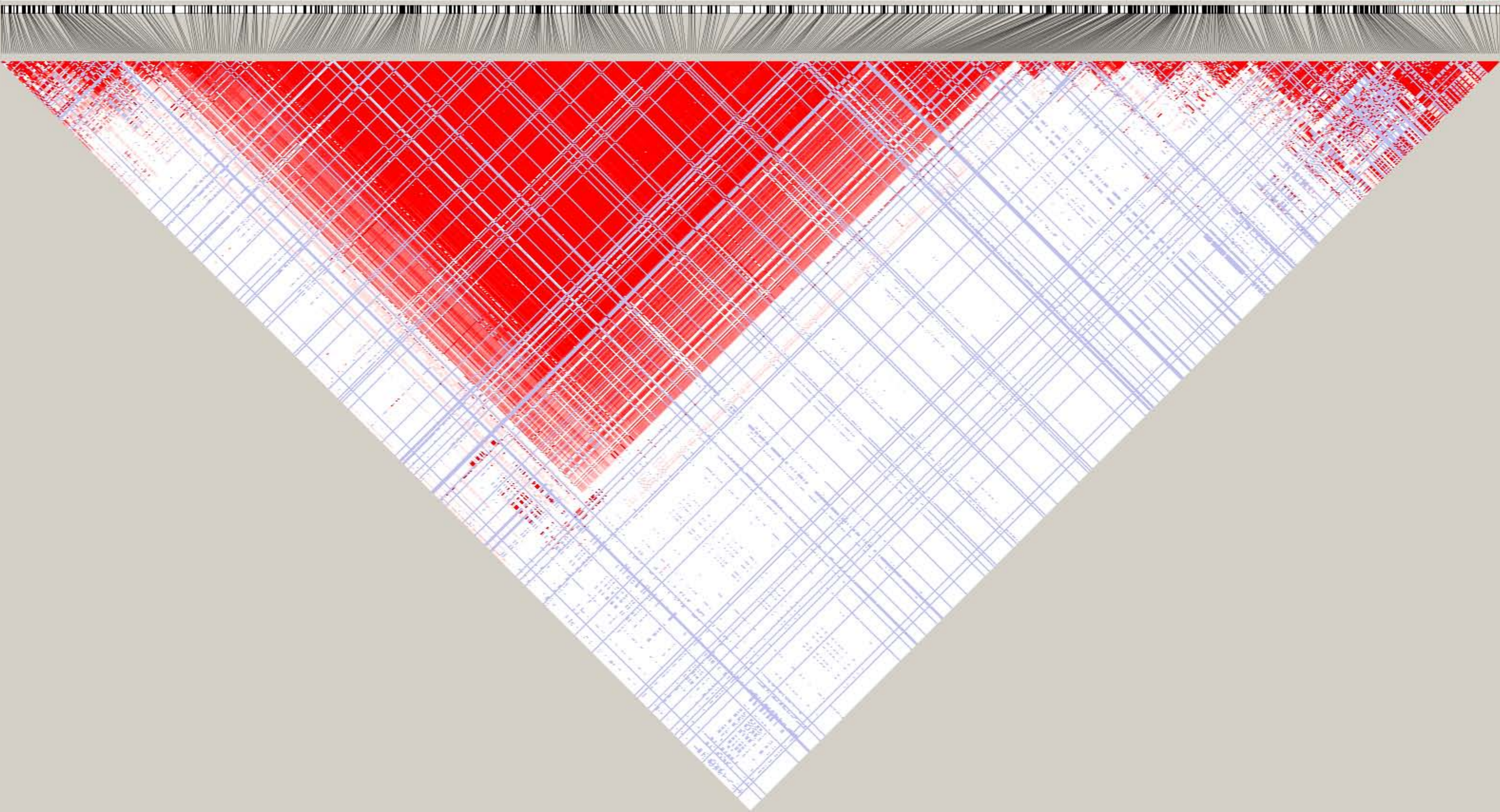

Supplement: Figure S2 — The LD plot for the region from 65,500 kb to 67,500 kb in chromosome 14 using CHB data (data with missing genotype less than 1% and no monomorphic site). (PDF) [file pone.0046603.s002.pdf]

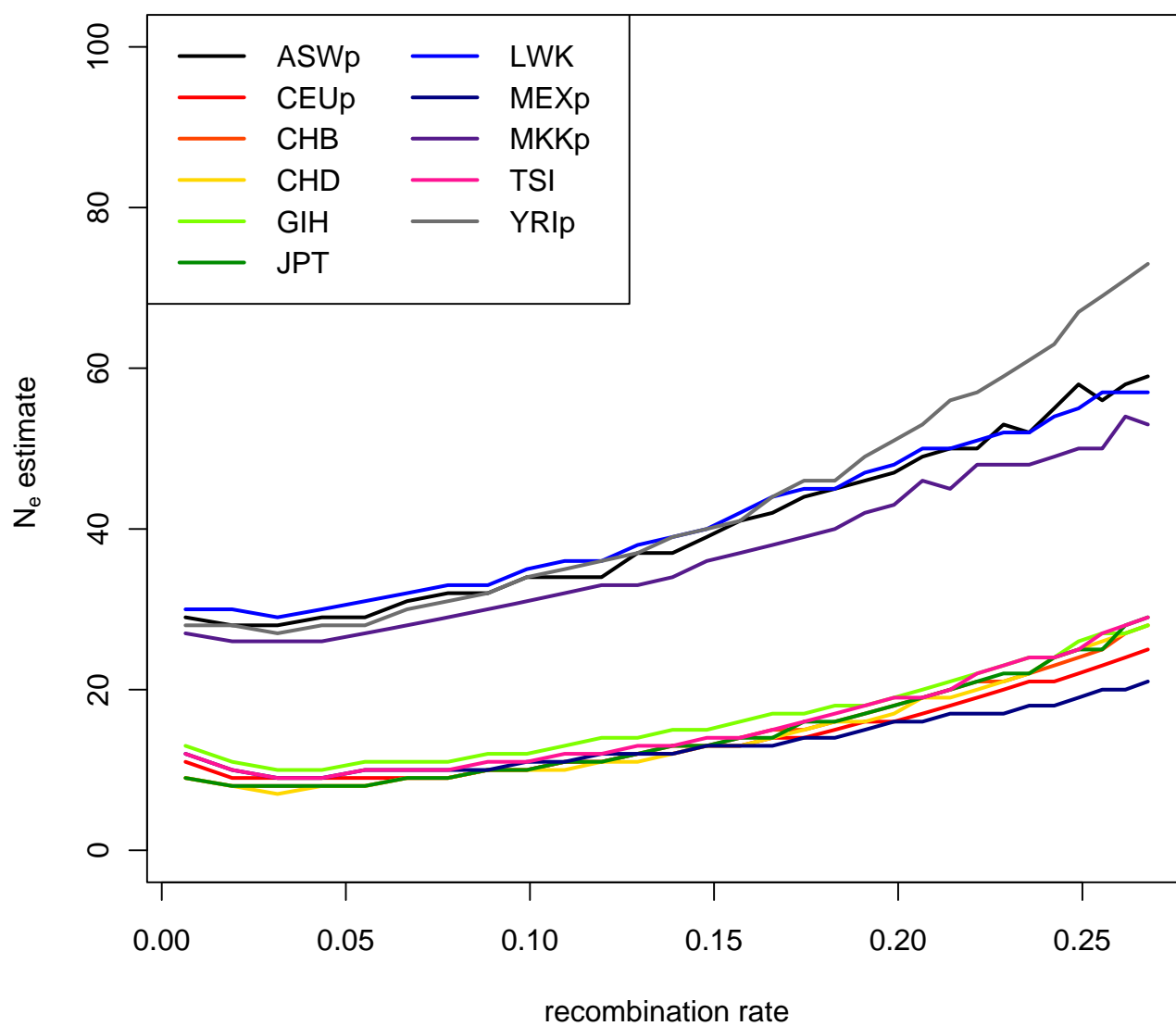

Supplement: Figure S4 — The enlargement of the last figure of Figure 4 ; Ne estimates of human population samples depending on recombination rates up to 0.3. (PDF) [file pone.0046603.s004.pdf]
