# Supplementary material for: Linkage Disequilibrium Decay and Past Population History in the Human Genome
Source: PLoS One. 2012 Oct 2;7(10):e46603. doi: 10.1371/journal.pone.0046603 (PMC3462787; doi:10.1371/journal.pone.0046603)
Supplement: Figure S3 — The histogram of minor allele frequency for the region from 65,500 kb to 67,500 kb in chromosome 14. (PDF) [file pone.0046603.s003.pdf]

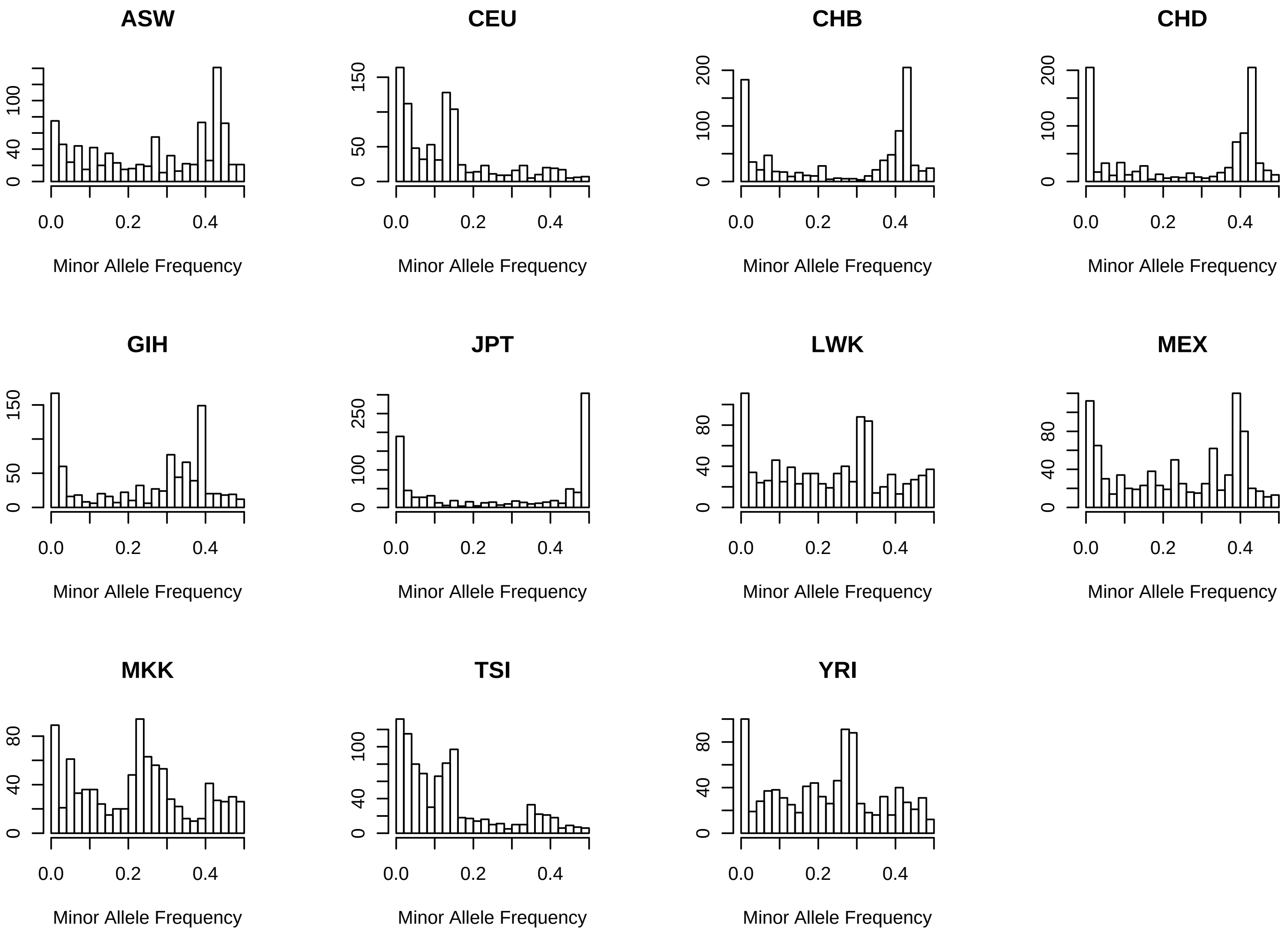
ASW
CEU
CHB
CHD
GIH
JPT
LWK
MEX
MKK
TSI
YRI
Minor Allele Frequency
0.0
0.2
0.4
