# Supplementary material for: Linkage Disequilibrium Decay and Past Population History in the Human Genome
Source: PLoS One. 2012 Oct 2;7(10):e46603. doi: 10.1371/journal.pone.0046603 (PMC3462787; doi:10.1371/journal.pone.0046603)
Supplement: Table S2 — Mean squared errors of sampling from fixed r2 for various sampling sizes with 10,000 SNP pairs and a population size of 1000 (r2 o: the original r2 of a population; ss: sample size). (DOC) [file pone.0046603.s006.doc]

Table S2. Mean squared errors of sampling from fixed r^2^ for various sampling sizes with 10,000 SNP pairs and a population size of 1000 (r^2^_o_: the original r^2^ of a population; ss: sample size).

| r^2^_o_  ss | **100** | **200** | **500** | **1000** | **2000** |
| --- | --- | --- | --- | --- | --- |
| **1** | 1.70E-32 | 2.44E-32 | 2.22E-33 | 2.47E-32 | 2.46E-32 |
| **0.81** | 3.25E-03 | 1.64E-03 | 6.77E-04 | 3.33E-04 | 1.81E-04 |
| **0.64** | 5.28E-03 | 2.67E-03 | 1.10E-03 | 5.69E-04 | 3.17E-04 |
| **0.49** | 6.01E-03 | 3.08E-03 | 1.33E-03 | 6.95E-04 | 4.00E-04 |
| **0.36** | 6.08E-03 | 3.17E-03 | 1.37E-03 | 7.45E-04 | 4.49E-04 |
| **0.25** | 5.41E-03 | 2.74E-03 | 1.24E-03 | 6.86E-04 | 4.25E-04 |
| **0.16** | 4.31E-03 | 2.26E-03 | 9.85E-04 | 5.85E-04 | 3.66E-04 |
| **0.09** | 2.95E-03 | 1.51E-03 | 6.86E-04 | 4.01E-04 | 2.56E-04 |
| **0.04** | 1.61E-03 | 7.98E-04 | 3.62E-04 | 2.09E-04 | 1.39E-04 |
| **0.01** | 5.97E-04 | 2.62E-04 | 1.03E-04 | 6.01E-05 | 3.91E-05 |
| **0** | 2.31E-04 | 6.35E-05 | 1.22E-05 | 4.63E-06 | 2.09E-06 |
